# Expression of glucocorticoid receptor and HDACs in airway smooth muscle cells is associated with response to steroids in COPD

**DOI:** 10.1186/s12931-024-02769-3

**Published:** 2024-05-29

**Authors:** Liang Zhou, Michael Roth, Eleni Papakonstantinou, Michael Tamm, Daiana Stolz

**Affiliations:** 1 Department of Clinical Research, University Hospital Basel and University of Basel, Basel, Switzerland; 2 Clinic of Respiratory Medicine and Pulmonary Cell Research, University Hospital Basel, Basel, Switzerland; 3 Clinic of Respiratory Medicine, Medical Center-University of Freiburg, Freiburg, Germany; 4 Faculty of Medicine, University of Freiburg, Freiburg, Germany

**Keywords:** Chronic obstructive pulmonary disease, Histone deacetylases, Glucocorticoid sensitivity, Glucocorticoid receptor, Airway smooth muscle cells

## Abstract

**Background:**

Steroid insensitivity in Chronic Obstructive Pulmonary Disease (COPD) presents a problem for controlling the chronic inflammation of the airways. The glucocorticoid receptor (GR) mediates the intracellular signaling of inhaled corticosteroids (ICS) by interacting with transcription factors and histone deacetylases (HDACs). The aim of this study was to assess if COPD patients’ response to ICS in vivo, may be associated with the expression of GR, the complex of GR with transcription factors, and the expression of various HDACs in vitro.

**Methods:**

Primary airway smooth muscle cells (ASMC) were established from endobronchial biopsies obtained from patients with asthma (*n* = 10), patients with COPD (*n* = 10) and subjects that underwent diagnostic bronchoscopy without pathological findings and served as controls (*n* = 6). ASMC were also established from 18 COPD patients, 10 responders and 8 non-responders to ICS, who participated in the HISTORIC study, an investigator-initiated and driven clinical trial that proved the hypothesis that COPD patients with high ASMC in their endobronchial biopsies respond better to ICS than patients with low ASMC. Expression of GR and its isoforms GRα and GRβ and HDACs was investigated in primary ASMC in the absence or in the presence of dexamethasone (10^− 8^M) by western blotting. The complex formation of GR with transcription factors was assessed by co-immunoprecipitation.

**Results:**

Expression of GR and its isoform GRα but not GRβ was significantly reduced in ASMC from COPD patients as compared to controls. There were no significant differences in the expression of GR, GRα and GRβ between responders and non-responders to ICS. However, treatment with dexamethasone upregulated the expression of total GR (*p* = 0.004) and GRα (*p* = 0.005) after 30 min in responders but not in non-responders. Τhe formation of the complex GR-c-Jun was increased 60 min after treatment with dexamethasone only in responders who exhibited significantly lower expression of HDAC3 (*p* = 0.005) and HDAC5 (*p* < 0.0001) as compared to non-responders.

**Conclusions:**

These data suggest that ASMC from COPD patients who do not respond to treatment with ICS, are characterized by reduced GR-c-Jun complex formation and increased expression of HDAC3 and HDAC5.

**Trial registration:**

ISRCTN11017699 (Registration date: 15/11/2016).

**Supplementary Information:**

The online version contains supplementary material available at 10.1186/s12931-024-02769-3.

## Background

Chronic Obstructive Pulmonary Disease (COPD) is a heterogeneous lung condition characterized by chronic respiratory symptoms such as dyspnea, cough, expectoration, and exacerbations. COPD cannot be cured, and only a limited symptom control can be achieved. Although inhaled glucocorticoids (ICS) are an effective therapy to control inflammation in asthma, the responsiveness and long-term (> 3 years) safety of ICS for COPD patients are unclear and requires further investigation [1, 2]. In vitro data suggest that COPD-associated inflammation is less responsive to ICS than asthmatic inflammation [3]. In COPD, ICS therapy alone cannot slow the long-term decline of lung function, which is reflected by continuous reduction of forced exhalation volume over 1 s (FEV1), nor did ICS therapy improve quality of life of COPD patients [4]. The cause of this lack of response to ICS is not well understood and presents a major problem to effectively treat lung malfunction in COPD patients.

Regarding the molecular biological action, ICS diffuse into the airway tissue where they bind and activate the cytosolic glucocorticoid receptor (GR), which subsequently is transported into the nucleus [5, 6]. One reason for steroid insensitivity is the existence of various isoforms of GR [7]. The two major GR-isoforms are GRα and GRβ but the anti-inflammatory effect of steroids is mainly mediated by the GRα isoform. The action of GRα can be hampered by GRβ, which acts as a dominant inhibitor [8, 9]. The function of GR can also be affected by the interaction with other transcription factors such as NF-κB, and the c-Jun sub-unit of AP-1 [10, 11]. Such interactions of the GR with other transcription factors can either reduce or enhance ICS sensitivity of different cell types in other diseases [12] but has not been reported for COPD.

Histone deacetylases (HDACs) are enzymes that remove acetyl groups from hyperacetylated histones, they counteract histone acetyltransferases (HATs), and thereby return histones to their basal state [13, 14]. HDAC expression and function can be affected by cigarette smoke and thus are of interest for the development of CODP pathologies [15, 16]. An important anti-inflammatory mechanism of the active GR is the recruitment of HDAC2 to activated inflammatory genes, which removes acetyl-groups from lysine and thereby switches off their expression [17]. Furthermore, HDAC2 suppressed IL17A-mediated airway remodeling in COPD, which is a main feature of COPD pathogenesis [18]. Therefore, reduced HDAC2 expression may account for low ICS response in subjects with COPD [19]. HDAC3 is a positive regulator of IL-1-induced gene expression [20]. However, it was reported that HDAC3 has cell-type/differentiation-specific stimulating and suppressive effects in macrophages in regard to TNFα and IL-6 secretion [21]. Lysine deacetylation by HDAC3 stimulated IκBα binding, which promoted NF-κB export from the nucleus to the cytoplasm, due to acetylation of NF-κB on different residues [22]. HDAC5 reduced the inflammatory response in lung tissue through NF-κB pathway [23, 24], and was rapidly reduced by TNF or IL-1β in rheumatoid arthritis [25]. HDAC8 was associated with the expression of smooth muscle actin in the cytoskeleton and may regulate the contractile capacity of smooth muscle cells [26]. Therefore, disrupting acetylation may result in abnormal gene expression that contribute to the pathogenesis of COPD and may control the responsiveness to ICS.

Airway smooth muscle cells (ASMCs) determine airway structure and function on different levels [27]. They have been indicated to play a crucial role in inflammation-related airway wall remodeling in COPD [28], and their response to damage by cigarette smoke might be the cause of increased proliferation and thus, airway wall thickening [29]. While the driving force of tissue remodeling by ASMC has been postulated earlier [30], the role of the GR and HDACs in this cell type has not been deeply investigated.

The aim of the present study was to utilize primary ASMC obtained from COPD patients who participated in the HISTORIC study to investigate whether the response of COPD patients to ICS in vivo, might be correlated with the expression levels of GR, the complex of GR with transcription factors, and the expression of various HDACs in vitro. Our hypothesis posits that there exists a variance in the expression levels of GR, the GR-transcription factor complex, and HDACs in ASMC among COPD patients who exhibit responsiveness to ICS versus those who do not respond.

## Methods

### Primary airway cells

Primary ASMC were established, as described earlier [31]. The first set of experiments was performed to generate the basis of the study and the cells were isolated from endobronchial biopsies obtained from patients with asthma (*n* = 7), patients with COPD (*n* = 10) and subjects that underwent diagnostic bronchoscopy without pathological findings and served as controls (*n* = 6).

A second set of ASMC was also established from endobronchial biopsies obtained from 18 COPD patients who participated in the HISTORIC study, an investigator-initiated and –driven, double-blind, randomized, placebo-controlled trial that tested the hypothesis that COPD patients with high ASMC in endobronchial biopsies respond better to corticosteroids compared with patients with low ASMC [32].

In the HISTORIC study, 190 COPD patients, Global Initiative for Chronic Obstructive Lung Disease Stage B–D, underwent bronchoscopy with endobronchial biopsy. Patients were divided into groups A and B, with high ASMC area (> 20% of the bronchial tissue area) and low ASMC area (⩽20% of the bronchial tissue area), respectively, and followed a run-in period of 6 weeks on open-label triple inhaled therapy with aclidinium (ACL)/formoterol (FOR)/budesonide (BUD) (400/12/400 µg twice daily). Subsequently, patients were randomised to receive either ACL/FOR/BUD or ACL/FOR/placebo and followed for 12 months. The primary end-point of the study was the difference in post-bronchodilator FEV1 over 12 months between patients with low ASMC and high ASMC receiving or not receiving ICS. At the end of the study patients who were receiving ACL/FOR/BUD were characterized as responders if they had an improvement if FEV1 or as non-responders if they had a decline in FEV1 [32].The study was approved by the Institution Review Board (EKNZ 2016-6-01880) and was registered with ISRCTN registry (ISRCTN11017699).

In the present study we analyzed primary ASMC that were established from endobronchial biopsies from 10 patients who responded to ICS treatment (responders) and 8 patients who did not respond to ICS treatment (non-responders). Among these 16 COPD patients 6 belonged to the group of high ASMC and 10 patients belonged to the group of low ASMC.

### Treatment of cells with steroids

Primary ASMC established from patients with COPD (*N* = 16), 8 responders and 8 non-responders to ICS treatment, as well as from controls (*n* = 5) were treated with 10^-8^M dexamethasone (Dex, #D4902-25MG, Sigma-Aldrich, St Louis, USA). The effect of dexamethasone on GR expression and localization was evaluated by western blot and immunofluorescence staining.

### Western blot

For Western-blots, cells were lysed in RIPA buffer (#SLCD5849, Sigma, USA) and the protein concentration of each sample was determined by BCA protein assay kit (#XI357440, ThermoFisher Scientific, Waltham, USA). The protein concentration was adjusted to 20 µg of total protein which were denatured (10 min, 95^o^C), and applied to electrophoresis was for size fractionation (110 V, open Amp, 50 min, at 4^o^C) in a 4–12% SDS–PAGE (#M41212, GeneScript, Piscataway, USA). Proteins were then transferred onto a nitro-cellulose membrane (#88,018, ThermoFisher, USA) by heat-accelerated capillary transfer and over-night incubation at 50^o^C. Primary antibodies were applied overnight at 4^o^C, followed by visualization with secondary, horse radish labeled antibodies. The following antibodies were used: c-Jun (1:1000, Abcam ab40766); NF-kB p65 (1:400, Cell Signaling D14E12); GR (1:1000, Abcam ab183127); HDAC2 (1:2000, Abcam ab219053); HDAC3 (1:500, Abcam ab32369); HDAC5 (1:1000, Abcam ab55403); HDAC8 (1:1000, Invitrogen PA5-83916); GAPDH (1:1000, Abcam ab181602); α-tubulin (1:1000,R&D systems MAB9344); Anti-Rabbit IgG (1:2000, Sigma A9169-2ML); Anti-Mouse IgG (1:2000, Sigma A9917-1ML).

Blots were then quantified by the Image J (1.53 version).

### Immunofluorescence staining

In order to determine the activation of the GR by dexamethasone (1 × 10^− 8^M), we performed immunofluorescence microscopy and sub-sequent image analysis for nuclear staining of the GR which presented as clearly localized green/turquois color.

For immuno-fluorescence analysis, cells were grown in 8-well chamber-slides, were treated with different conditions, were washed with phosphate buffered saline (PBS), and then fixed in 4% formaldehyde. Unspecific binding was prevented by 1 h incubation with 2% bovine serum albumin in PBS before the primary antibody for GR (1:500, Abcam ab183127) was applied overnight at 4^o^C. Following 3 washes with PBS, slides were incubated with Alexa Fluor™ 488 (#A-11,008, Thermo Scientific, USA) for 30 min and washed again with PBS. Nuclei were stained by 4’-6-diamidino-2-phenylindole, dihydrochloride (DAPI). Images were acquired by ECLIPSE Ti2 (Nikon, Tokyo, Japan) and documented by imaging software NIS-Elements (Nikon, Japan).

### Co-immunoprecipitation

Cells were grown in 25 cm flasks to confluence, before being serum deprived for 24 h, and afterwards been treated with dexamethasone(1 × 10^-8^M) for 24 h. Co-immunoprecipitation was performed using the Immunoprecipitation kit (*#*ab206996, Abcam, Cambridge, U.K.). according to instructions. In brief, ASMC were lysed in non-denaturing lysis buffer containing: 0.1% SDS, 1% NP40 and 0.5% deoxycholate and cells were scrapped off after 10 min. The collected protein solution 100 µg) was incubated for over-night (4^o^C) with one of the primary antibodies (see below). A non-specific antibody served as control The protein/antibody mix was then incubated with binding sepharose beads, provided by the kit for 1 h (4^o^C); 5% bovine serum albumin (in PBS) was used to block non-specific binding. Beads were collected and washed three times with blocking buffer by centrifugation (2000 x g, 2 min, 4^o^C). The antibody/antigen complex was eluted in add 2X SDS-PAGE loading buffer by boiling for 5 min. The beads were separated by centrifugation (2000 x g, 2 min, 4^o^C) and the supernatant was collected to analyse the protein complexes by Western-blotting directly (denaturing condition) as well as by low-pH, non-denaturing condition. The latter method was performed by the addition of 40 µL low pH glycine buffer (100 mM glycine/HCl, pH 2.5) for 10 min at room temperature, followed by centrifugation (2000 x g, 2 min, 4^o^C), before the pH was adjusted by adding 1/10 of the volume with 1 M Tris/HCl (pH 8.5) to neutralize the pH. Protein analysis was performed by Western-blotting.

Primary antibodies used for Co-Immunoprecipitation were: GR (1:100, Abcam D6H2L); c-Jun (1:1000, Abcam ab40766); NF-kB p65 (1:400, Cell Signalling D14E12). Protein bands were visualized by Western-blotting as described above.

### Statistical analysis

GraphPad Prism 9.0 software was used for data analysis. Data are represented as mean ± SEM. Statistical analysis was performed by Student’s t-test or one-way ANOVA test. The data were presented as mean ± SEM of the results from at least three independent experiments. P-value < 0.05 was considered statistically significant.

## Results

### Expression of total GR, GRα and GRβ in primary ASMC

**Fig. 1:**
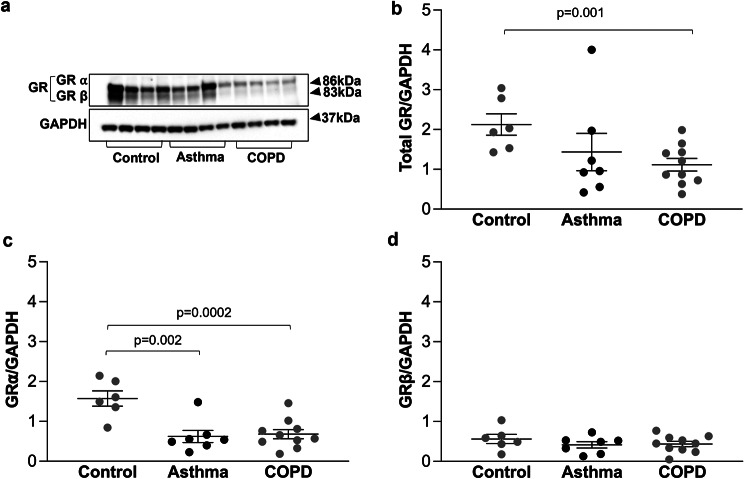
Expression of total GR, GRα and GRβ in primary ASMC. (**a**) Representative Western-blots of the expression of total GR, GRα and GRβ in primary ASMC established from controls (*n* = 6), patients with asthma (*n* = 7 ) and patients with COPD (*n* = 10). **b**-**d** Quantitation of the western blots performed by Image J. Expression of total GR represents the sum of the expression of GRα and GRβ isoforms. Error bars represent mean ± SEM

Total GR, GRα and GRβ protein expression was determined in primary ASMC from patients with asthma, COPD and from controls by western blot (Fig. 1a). Quantitation of the blots revealed that total GR expression was significantly lower in patients with COPD (*p* = 0.001) as compared to controls (Fig. 1b). Furthermore, the expression of GRα isoform was significantly lower in patients with asthma (*p* = 0.002) and COPD (*p* = 0.0002), as compared to controls (Fig. 1c) but there was no difference in the expression of GRβ between controls and patients with asthma or COPD (Fig. 1d).

### Expression of total GR, GRα and GRβ in primary ASMC from COPD patients who responded or did not respond to treatment with ICS

**Fig. 2:**
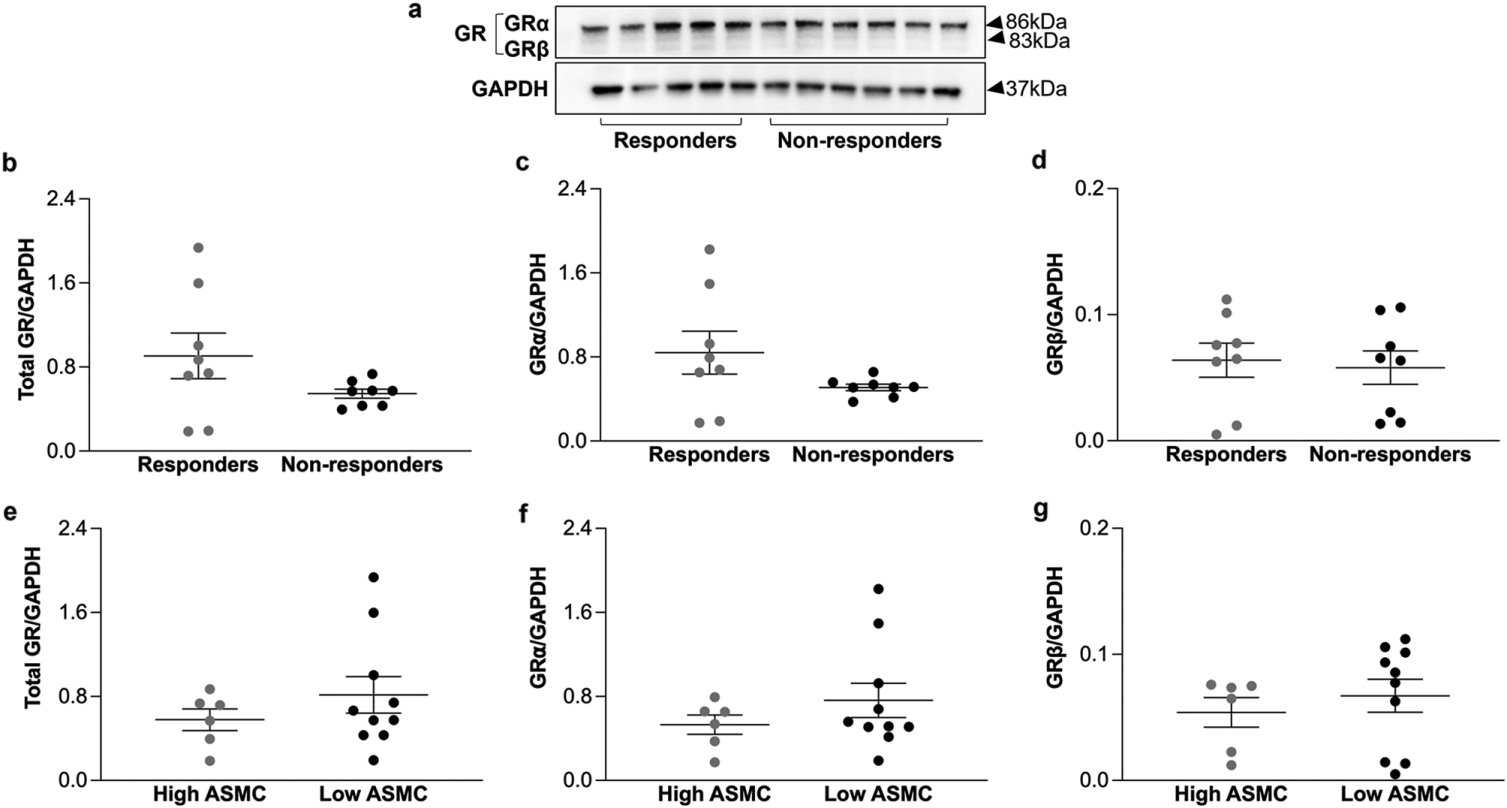
Expression of total GR, GRα and GRβ in primary ASMC from COPD patients. (**a**) Representative Western-blots of the expression of total GR, GRα and GRβ in primary ASMC from 8 patients responded to treatment with ICS (responders) and 8 patients did not respond to treatment with ICS (non-responders). (**b**-**g**) Quantitation of the western blots performed by Image J. Among the 16 COPD patients, 6 patients had high ASMC (> 20% of the bronchial tissue area) and 10 patients had low ASMC (⩽20% of the bronchial tissue area), in their endobronchial biopsy (**e, f, g**). Expression of total GR represents the sum of the expression of GRα and GRβ isoforms. Error bars represent mean ± SEM. Statistical analysis revealed no significant differences between the groups

We further examined the expression of total GR, GRα and GRβ in primary ASMC established from endobronchial biopsies from 19 COPD patients who participated in the HISTORIC study by western blot (Fig. 2a). Among these patients, 9 patients responded to treatment with ICS and 10 patients did not respond to treatment with ICS. Quantitation of the western blots revealed that there was no difference in the expression of total GR (Fig. 2b), GRα (Fig. 2c) or GRβ (Fig. 2d) between responders and non-responders to ICS. The expression of total GR, GRα and GRβ was also similar between patients who had high amount of ASMC (> 20% of the bronchial tissue area) and low ASMC area (⩽20% of the bronchial tissue area), in their endobronchial biopsies (Fig. 2e and g).

### Effect of dexamethasone on the expression and activation of GR in primary ASMC

**Fig. 3:**
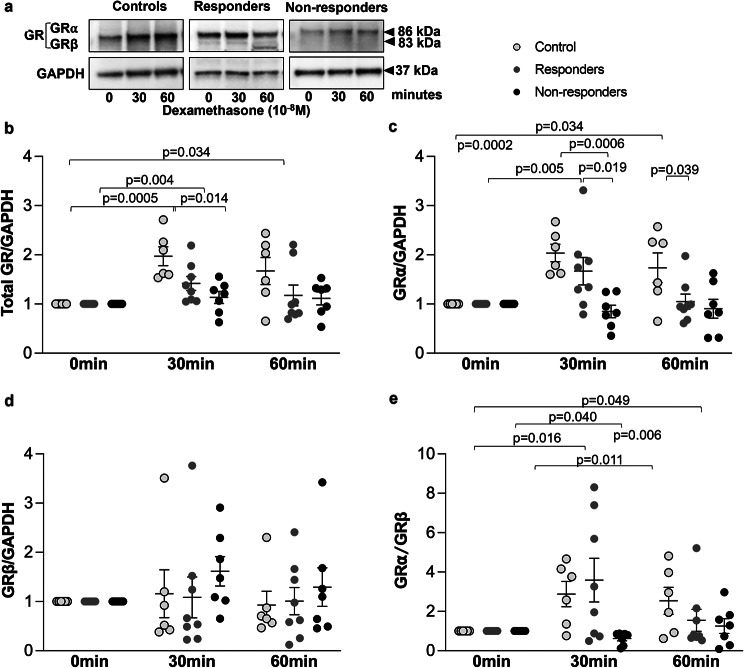
Expression of total GR, GRα and GRβ in response to dexamethasone within 60 min. (**a**) Representative Western-blots of the effect of dexamethasone (10^-8^M) on the expression of total GR, GRα and GRβ in primary ASMC from controls (*n* = 6), COPD patients who responded to treatment with ICS (*n* = 8) and COPD patients who did not response to treatment with ICS (*n* = 7). (**b-e**) Quantitation of the western blots performed by Image J. Expression of total GR represents the sum of the expression of GRα and GRβ isoforms. Results are expressed as fold change to time 0. Error bars represent mean ± SEM

The effect of dexamethasone (10^− 8^ M), on the expression of total GR, GRα and GRβ by primary ASMC established from controls and from COPD patients who responded or did not respond to ICS was also examined (Fig. 3a). Dexamethasone upregulated the expression of total GR after 30 min (*p* = 0.0005) and 60 min (*p* = 0.034) in ASMC from controls and after 30 min (*p* = 0.004) in ASMC from responders, (Fig. 3b) but had no effect on total GR expression in non-responders (Fig. 3a and b).

Similarly, the expression of GRα was increased in response to dexamethasone in ASMC from controls after 30 min (*p* = 0.0002) and 60 min (*p* = 0.034) and after 30 min in ASMC from responders (*p* = 0.005) (Fig. 3c) whereas dexamethasone had no effect on the expression of GRβ (Fig. 3d). The ratio of GRα/GRβ was increased in response to dexamethasone in ASMC from controls after 30 min (*p* = 0.016) and 60 min (*p* = 0.049) and after 30 min in ASMC from responders (*p* = 0.040) but there was no effect for non-responders (Fig. 3e).

**Fig. 4:**
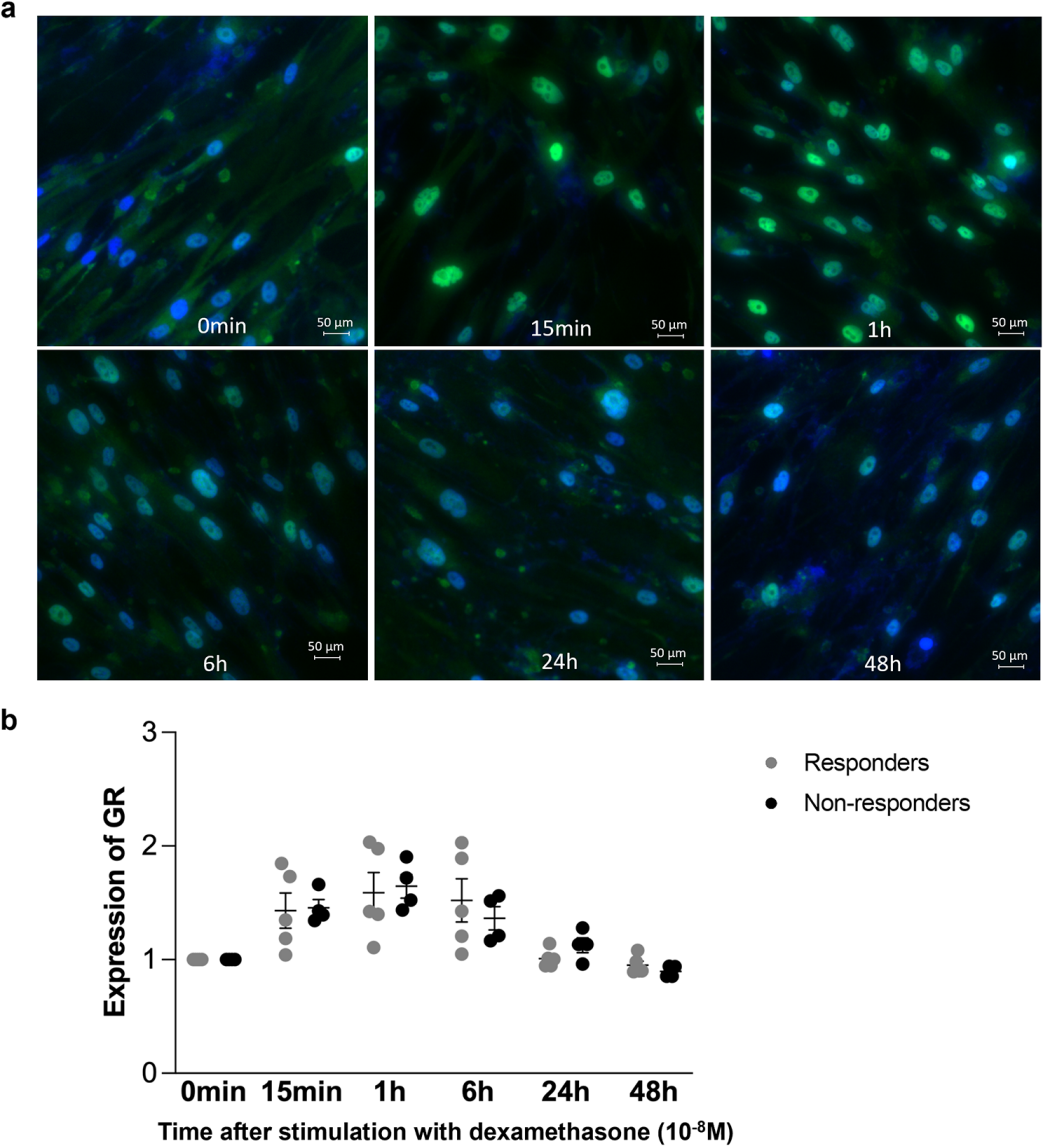
Activation of GR in primary ASMC in response to dexamethasone within 48 h. The activation of the GR (green/turqoise) was determined by its translocation into the nucleus (blue). (**a**) Representative immunofluorescence photographs showing the activation of GR and its translocation into the nucleus in response to dexamethasone (1 × 10^-8^M). Photographs with larger cell area are provided in supplemantary Fig. 1. (**b**) Quantitation of the staining in ASMC from COPD patients, 5 responders to ICS and 4 non-responders to ICS. Images were acquired by ECLIPSE Ti2 (Nikon, Tokyo, Japan) and documented by imaging software NIS-Elements (Nikon, Japan). Results represent the expression of GR measured by the intensity of green colour as fold change to time 0. Error bars represent mean ± SEM. Green: GR; Blue: nuclei

We further examined the translocation of GR from the cytosol into the nucleus as the result of GR activation after stimulation with dexamethasone (1 × 10^-8^ M). Immunofluorescence microscopy and sub-sequent image analysis showed that the nuclear staining of the GR presented as a clearly localized green/turquois color (Fig. 4a; a larger cell area is presented in supplementary Fig. 1). As shown in Fig. 4a, the intensity of the signal for the nuclear GR increased within 15 min after the addition of dexamethasone, it peaked at 1 h, and then it decreased. After 24 h, the total GR expression level was similar to the level before dexamethasone treatment and then further decreased over 48 h (Fig. 4a). Quantitation of the staining revealed that the activation of GR in response to dexamethasone was similar in ASMC from responders to ICS and from non-responders to ICS (Fig. 4b).

### Complex of GR with transcription factors

**Fig. 5:**
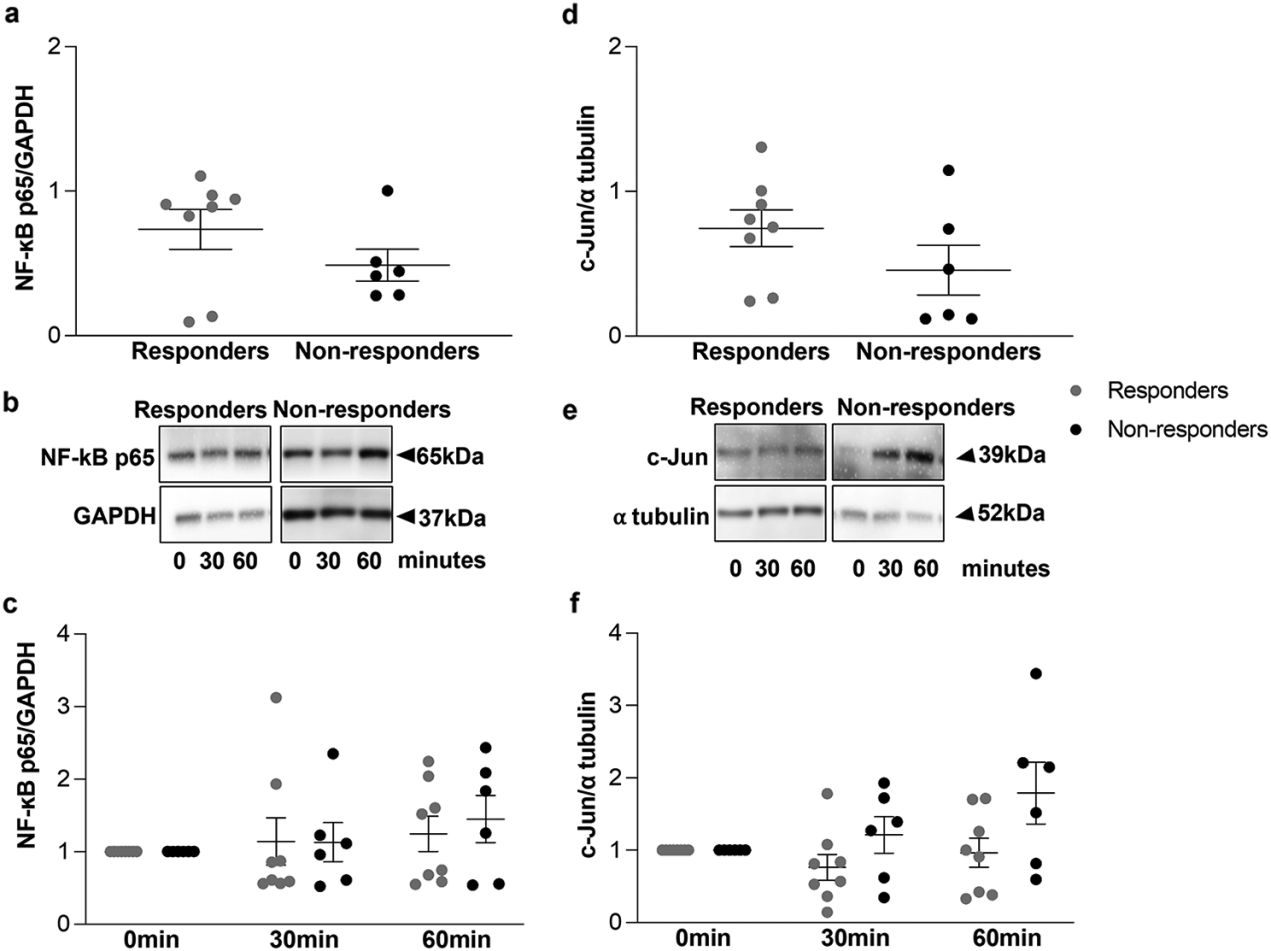
Expression of transcription factors in primary ASMC in response to dexamethasone. Expression of NF-kB (**a**) and c-Jun (**d**) in primary ASMC from responders and non-responders to ICS. Results represent the quantitation of respective western blots from 8 responders and 6 non-responders. Error bars represent mean ± SEM. Representative Western-blots of the effect of dexamethasone (10^-8^M) on the expression of total NF-kB (**b**) and c-Jun (**e**) in primary ASMC from 8 responders and 6 non-responders to ICS. (**c** and **f**) Quantitation of the western blots performed by Image J. Results represent fold change to time 0. Error bars represent mean ± SEM

Expression levels of NF-kB and c-Jun was similar in responders and non-responders (Fig. 5a and d). Treatment of ASMC with dexamethasone did not alter the expression level of the proinflammatory transcription factors NF-κB (Fig. 5b and c) and c-Jun (Fig. 5e and f) neither in ASMC from responders to ICS nor in ASMC from non-responders to ICS.

**Fig. 6:**
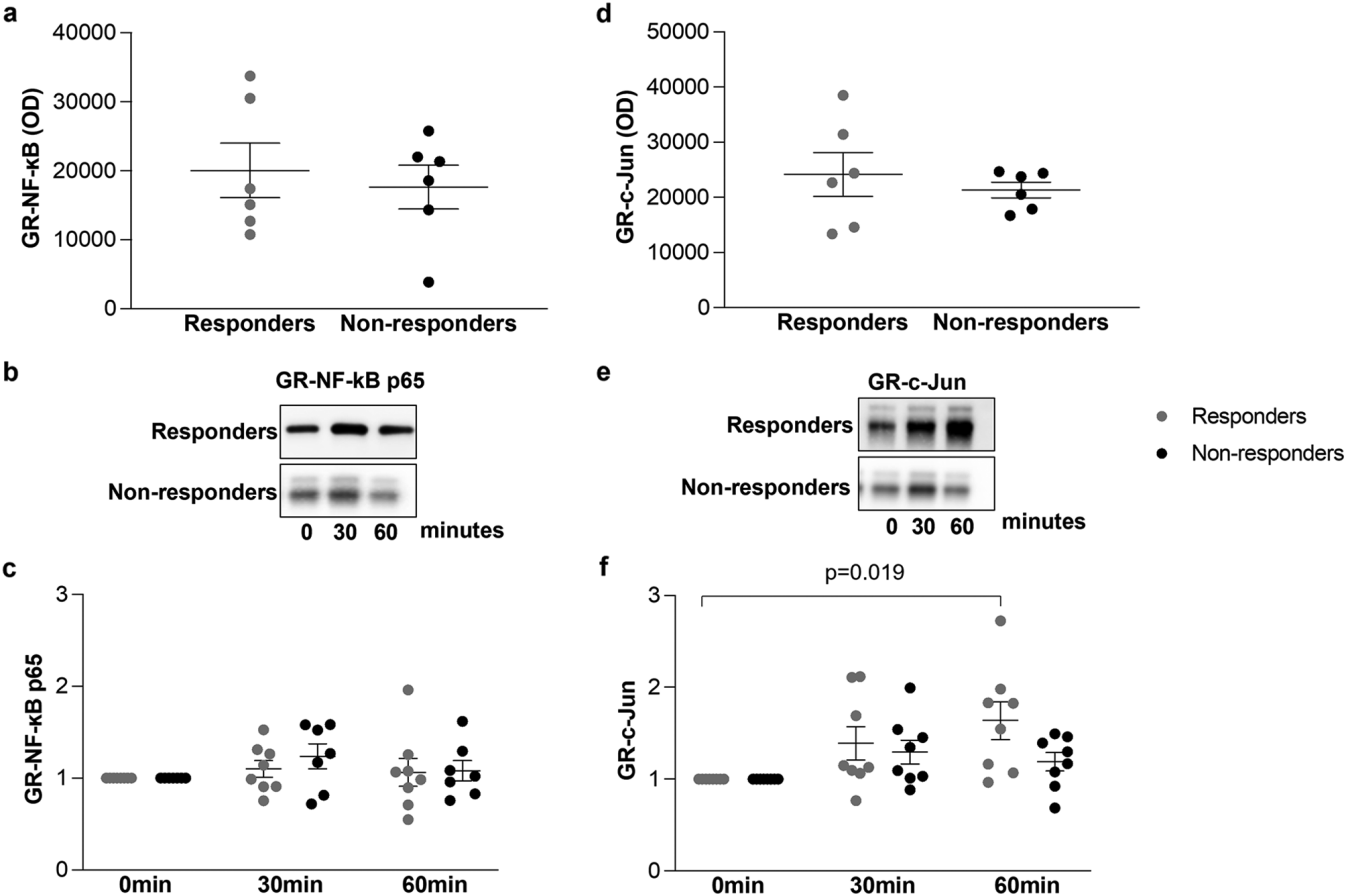
Complex of GR and transcription factors in primary ASMCs in response to dexamethasone within 60 min. Complex of GR and NF-kB (**a**) and GR and c-Jun (**d**) transcription factors in primary ASMC from COPD patients who responded or did not respond to ICS assessed by co-immunoprecipitation. Results represent the quantitation of respective western blots from 6 responders and 6 non-responders. Error bars represent mean ± SEM. Representative Western-blots of the effect of dexamethasone (1 × 10^-8^M) on the expression of GR-NF-kB complex (**b**) and GR-c-Jun complex (**e**) in primary ASMC from 6 responders and 6 non-responders to ICS. **c** and **f** Quantitation of the western blots performed by Image J. Dots represent fold change to time 0. Error bars represent mean ± SEM

Co-immunoprecipitation revealed that complex formation of GR with NF-κB and c-Jun was similar in responders and non-responders to ICS (Fig. 6a and d). Treatment of ASMC with dexamethasone had no effect on the complex formation of GR with NF-kB neither in responders nor in non-responders to ICS (Fig. 6c). However, the complex of GR with c-Jun was significantly increased after 60 min in response to dexamethasone but only in ASMC from responders to ICS (*p* = 0.019) and not in ASMC from non-responders to ICS (Fig. 6f).

### Expression of HDACs in primary ASMC

**Fig. 7:**
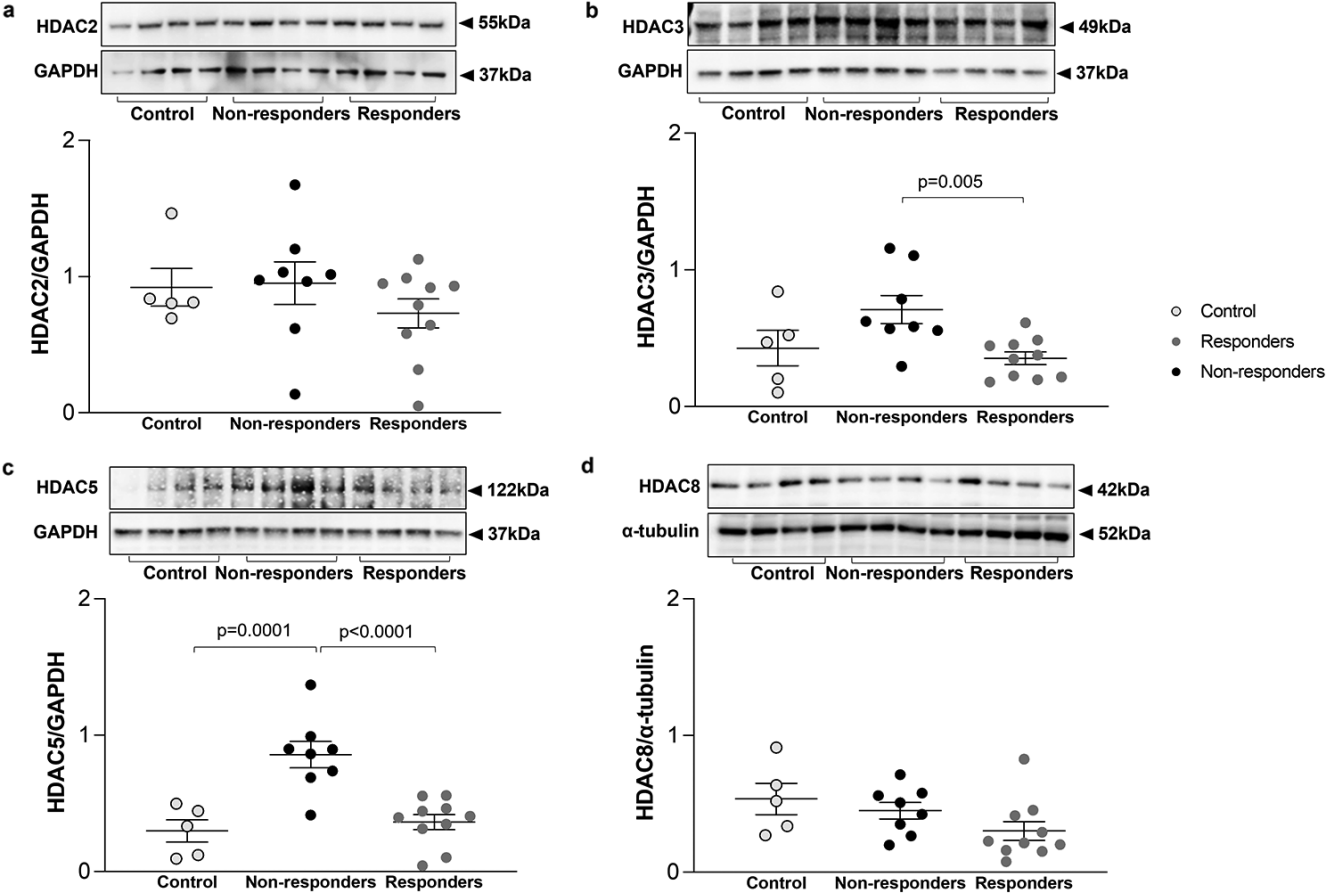
Expression of HDACs in primary ASMC from controls and from COPD patients. Representative western blots for the expression of HDAC2 (**a**), HDAC3 (**b**), HDAC5 (**c**) and HDAC8 (**d**) in primary ASMC from controls (*n* = 5) and patients with COPD who responded (*n* = 10) or did not respond to ICS (*n* = 8). GAPDH was used as loading control for large molecular weight HDACs and α-tubulin for small molecular weight HADC8. Quantitation of the western blots performed by Image J. Error bars represent mean ± SEM

The expression of HADC2, HDAC3, HDAC5, and HDAC8 was determined by Western-blotting in primary ASMC from controls and from COPD patients who responded or who did not respond to ICS. There were no significant differences in the expression of HDAC2 (Fig. 7a) and HDAC8 (Fig. 7d) between controls and responders or non-responders. However, the expression of HDAC3 (Fig. 7b) and HDAC5 (Fig. 7c) was significantly lower in ASMC from responders as compared to non-responders (*p* < 0.005 and *p* < 0.001, respectively).

## Discussion

The aim of this study was to assess if the in vitro expression of GR and HDACs in primary ASMC from COPD patients is associated with their response to treatment with ICS in vivo. We established primary ASMC from endobronchial biopsies obtained from COPD patients who were included in the HISTORIC study and, after 12 months follow-up, were well characterized for their response to treatment with ICS. The expression of GR and its isoforms GRα and GRβ were similar between responders and non-responders. However, treatment with dexamethasone resulted in upregulation of GR and GRα only in responders and not in non-responders. Τhe formation of the complex GR-c-Jun was significantly increased 60 min after treatment with dexamethasone only in responders who exhibited significantly lower expression of HDAC3 and HDAC5 as compared to non-responders.

The cellular GR expression level has been correlated with glucocorticoids sensitivity [33]. Reduced GR number was found in peripheral blood cell and airway epithelial cells from patients with asthma and COPD with poor clinical response to ICS [34–36]. In contrast, the expression of GRβ, the dominant negative isoform, was found to be up-regulated in immunocytes and airway epithelial cells isolated from asthma patients with poor responsiveness to ICS [37]. In this study, the expression level of total GR was significantly lower in ASMC from COPD patients as compared to controls. This maybe attributed rather to the lower expression of GRα isoform that was evident in ASMC from COPD and asthma patients and not to GRβ isoform the expression of which was similar among controls and patients with asthma and COPD.

Neither total GR nor GRα and GRβ expression levels were associated with COPD patients’ response to ICS. This finding is in line with an earlier reported failure to use the GR-isoform ratio for predicting patient’s response to steroid in young adults [38]. Even though the ratio of GR isoforms and their expression level was suggested to indicate patients’ response to steroids, including ICS [39], the use of GR expression level as an indicator of individual’s response to ICS is hindered by the plethora of isoforms and interactions, which can modify the GR’s anti-inflammatory and anti-proliferative actions in many levels [40]. Furthermore, it has to be noted that the function of GR may also vary between different bronchi location, and it may also be affected by the circadian rhythm of hormones [41].

Furthermore, the expression of GR in vivo can be altered by many environmental factors including cigarette smoke, the best characterized risk factor for COPD, which has been shown to reduce the expression of GR [10]. This effect of cigarette smoke was observed in a rat model during pregnancy, thereby setting the offspring to develop COPD-like symptoms later in life [42]. The mechanism behind this lasting, and probably inheritable effects of GR-expression in the context of COPD is not well understood and needs further investigation, similar to the assessment described in asthma [43].

Long-term treatment with glucocorticoids has been associated with reduced expression of GR by a feedback mechanism. The expression level of GRα in patients with systemic lupus erythematosus was decreased, after 2 weeks of dexamethasone treatment [44]. In respiratory epithelial cells GRα was also downregulated by steroids after 24 h [45]. In the present study, dexamethasone induced the expression of GR and GRα within 30 min in ASMCs from controls and from COPD patients who responded to treatment with ICS but not in COPD patients who did not respond to ICS. However, when we examined the translocation of GR from the cytosol into the nucleus there was no significant difference between responders and non-responders to ICS during a period of 48 h. Thus, the long-term effect of ICS needs to be further investigated.

The biological function of GR can be affected by the formation of complexes with transcription factors such as NF-κB, c-Jun sub-unit of AP-1, or STAT [10, 11]. In the present study, the complex formation of GR with c-Jun was increased in ASMC from COPD patients who responded to treatment with ICS but not in COPD patients who did not respond to ICS. In contrast, there was no induction of GR-NF-κB complex, which has been previously reported [12]. The GR-c-Jun complex formation may attenuate inflammation through reducing c-Jun’s coactivator activity on pro-inflammatory cytokine genes [46]. However, the GR-c-Jun complex has been shown to have a negative feedback effect on the expression of GR in isolated ASMC of patients receiving ICS therapy [47]. Furthermore, the GR-c-Jun complex may account for the reduced activation of pro-inflammatory protein encoding genes such as interleukin-1 through activation of dual specific phosphatase 1 (DUSP1) [48]. In an earlier study we showed that DUSP1 mediated the anti-inflammatory effect of heat shock protein 70 through the up-regulation of the GR in isolated human primary ASMC [49]. Thus, our data indicate that an increased complex of GR-c-Jun might improve the response to ICS in COPD responders, but further details of this mechanism need to be investigated.

Transcription factor activity and DNA binding are also linked to HDAC activity, which is affected by cigarette smoke as mentioned above [15, 16]. Moreover, a cell type specific expression of the different HDACs has also to be taken into consideration [48]. It has been shown that mRNA levels of HDAC2 and HDAC8 in peripheral lung tissue and macrophages from COPD patients were lower compared to normal subjects [50]. Furthermore, it has been shown that HDAC2 is reduced in alveolar macrophages from COPD patients and this reduction is correlated with corticosteroid insensitivity [51]. In the present study, however, the expression of HDAC2 and HDAC8 in ASMC was similar between controls and patients with COPD. Despite their effect in the lung, it is interesting to note that HDACs might affect the function of skeletal muscles with regards to the reduced breathing capacity of COPD patients [51]. However, caution has to be taken when interpreting in situ HDAC expression as it is modified by commonly prescribed drugs for chronic lung inflammation, such as steroids and long acting β2-agonists [52]. Together these observations suggest a cell-type specificity in HDAC expression or a restoration of HDAC synthesis capacity in ASMC from COPD patients under culture conditions. To delve deeper into this matter, further investigations employing immunohistochemistry on endobronchial biopsies from COPD patients, stratified based on their response to ICS, are warranted. These experiments would provide insights into whether the observed differences in HDAC expression are indeed cell-type specific or influenced by other factors such as the local microenvironment in vivo.

HDAC3 plays a key role in inflammation and its deficiency resulted in reduced inflammatory gene expression in macrophages [21, 53]. In this context, up-regulated HDAC3 expression induced endothelial-to-mesenchymal transition (EMT) in mice [54], which is a potential mechanism contributing to small-airway fibrosis, a main pathology in COPD [55]. In this study, HDAC3 levels were higher in ASMC from COPD patients who did not respond to ICS compared to COPD patients who responded to ICS. In line with this finding, it has been shown that GR-mediated actions are repressed via the GR-NCoR1-HDAC3 complex [56]. HDAC3 formed a repressive complex acting at the transcriptional start site of the GR gene and silenced GR expression [56]. These findings indicate that HDAC3 may be used to predict inflammatory levels and response to ICS.

In the present study we also show that the expression of HDAC5 was significantly higher in ASMC from COPD patients who did not respond to ICS as compared to COPD patients who responded to ICS or to controls. It has been shown that HDAC5 inhibitors abolished the glucocorticoid-induced down-regulation of GR in rats [57]. In another study, knock-down of HDAC5 reduced the production of pro-inflammatory cytokines such as TNFα [24]. This evidence may account for the observed relative higher expression of HDAC5 in ASMC from non-responders compared to responders or controls. Furthermore, HDAC3 and HDAC5 can bind to c-Jun [58, 59], and HDACs had epigenetic effects when they formed a complex with other transcription factor [60]. Thus, the link of GR-c-Jun complex and HDAC3 and HDAC5 need to be further investigated. Contrary to the above studies and our data reduced transcription of HDAC5 but not protein levels were reported in COPD patients [48]. In summary, the differential expression of the various HDACs might be linked to reduced steroid responsiveness as well as to a deregulated expression of the GR. However, the exact mechanism of both events remains to be studies. Additional studies aimed at silencing these HDACs would be valuable in further understanding their role in GR functions.

One limitation of our study include the limited number of available primary ASMC from responders and non-responders as well as the limited number of time points to study the GR-interacting proteins. A second limitation is the lack of establishing individual steroid response curves in our cell culture model. Similar problems have been described in other diseases [61]. At least we could show that neither GR or GR-isoform expression correlated with clinical steroid response or ASMC area with the chosen in vitro end-points such as translocation kinetics or efficiency. However, to our knowledge, this is the first study that involves primary ASMC from a very well characterized cohort with proven response to treatment with ICS after a randomized, placebo controlled clinical trial. As our study was centered on COPD, we did not incorporate data regarding GR function and dexamethasone sensitivity in ASMC from asthma patients, a facet that warrants further investigation and clarification.

In summary, the results of our study indicate that GR expression levels in primary ASMC are not associated with response to steroids, however, the complex formation of GR with c-Jun, or the expression of HDAC3 and HDAC5 might help to predict the response of COPD patients to ICS. The different expression of HDAC3 and HDAC5 could explain the difference regarding the formation of the GR-c-Jun complex in responders to ICS.

### Electronic supplementary material

Below is the link to the electronic supplementary material.

Supplementary Material 1

## Data Availability

No datasets were generated or analysed during the current study.

## Abbreviations

ACL: Aclidinium
ASMC: Airway smooth muscle cell
BUD: Budesonide
COPD: Chronic obstructive pulmonary disease
FEV1: Forced expiratory volume in 1 s
FOR: Formoterol
GR: Glucocorticoid receptor
HAT: Histone acetyltransferase
HDAC: Histone deacetylases
ICS: Inhaled corticosteroids
